# Parallelization of enumerating tree-like chemical compounds by breadth-first search order

**DOI:** 10.1186/1755-8794-8-S2-S15

**Published:** 2015-05-29

**Authors:** Morihiro Hayashida, Jira Jindalertudomdee, Yang Zhao, Tatsuya Akutsu

**Affiliations:** 1 Bioinformatics Center, Institute for Chemical Research, Kyoto University, 611-0011, Uji, Japan Full list of author information is available at the end of the article

## Abstract

Enumeration of chemical compounds greatly assists designing and finding new drugs, and determining chemical structures from mass spectrometry. In our previous study, we developed efficient algorithms, *BfsSimEnum *and *BfsMulEnum *for enumerating tree-like chemical compounds without and with multiple bonds, respectively. For many instances, our previously proposed algorithms were able to enumerate chemical structures faster than other existing methods.

Latest processors consist of multiple processing cores, and are able to execute many tasks at the same time. In this paper, we develop three parallelized algorithms *BfsEnumP1-3 *by modifying BfsSimEnum in simple manners to further reduce execution time. BfsSimEnum constructs a family tree in which each vertex denotes a molecular tree. BfsEnumP1-3 divide a set of vertices with some given depth of the family tree into several subsets, each of which is assigned to each processor.

For evaluation, we perform experiments for several instances with varying the division depth and the number of processors, and show that BfsEnumP1-3 are useful to reduce the execution time for enumeration of tree-like chemical compounds. In addition, we show that BfsEnumP3 achieves more than 80% parallelization efficiency using up to 11 processors, and reduce the execution time using 12 processors to about 1/10 of that by BfsSimEnum.

## Introduction

Enumerating chemical compounds assists designing drugs and determining chemical structures from mass spectrometry. Hence, algorithms and mathematical models for the enumeration have been developed. A chemical compound is often represented as a molecular graph for the enumeration, which is defined as a connected graph with vertices labeled by atomic symbols and multi-edges labeled by chemical bonds. Here, the degree of a vertex means the valence of the atom and the multiplicity of a multiedge means the bond type. Given chemical formula and some restrictions, chemical structures desired for a biological system are enumerated by constructing all distinct graph structures. MOLGEN has been developed over two decades [1,2], and becomes a popular enumeration tool. EnuMol enumerates tree-like chemical compounds, or molecular tree graphs, by depth-first search (DFS) order [3-5]. In our previous study, we developed efficient algorithms *BfsSimEnum *and *BfsMulEnum *for enumeration of tree-like chemical compounds by breadth-first search (BFS) order [6]. For many instances, execution times by BfsSimEnum and BfsMulEnum were shorter than or comparable to those by the DFS-type method.

Latest processors consist of multiple cores even for personal use, and are able to execute many tasks at the same time. For further reducing execution time and providing better enumeration tools using web servers and stand-alone systems, in this paper, we make use of parallel computing and propose three parallelized algorithms *BfsEnumP1-3 *by modifying BfsSimEnum, which enumerates molecular tree graphs without addition of multi-edges. BfsMulEnum receives the output of BfsSimEnum, and enumerates chemical compounds with multiple bonds by changing single edges to multi-edges so that the restrictions are satisfied.

BfsSimEnum constructs a family tree in which each vertex corresponds to a molecular tree and is generated by DFS order. Several parallel algorithms for depth-first search have been developed [7,8]. Freeman introduced parallel algorithms and combined them with randomized algorithms for performing a depth-first search of a given graph [9]. Rao and Kumar developed parallel algorithms for depth-first search [10] and applied their algorithms to depth-first branch-and-bound and iterative-deepening A* (IDA*) [11], in which some cost function is defined and the algorithm tries to find optimal solutions. In the search, the parts not leading to an optimal solution are eliminated. Each processor has a stack that stores state space to be searched because it searches in a depth-first manner. In their algorithm, if a processor receives a request from another processor, then it splits its own stack into two sets of states and transfers one, where equally split, called $\frac{1}{2}$-split, is considered to be ideal in the paper. These parallel algorithms, however, are still complicated and require extra communication between processors and extra processes for polling. Our proposed algorithms BfsEnumP1-3 are simple, each of which divides a set of vertices at some given depth *d *of a family tree into several subsets, and each subset is assigned to one processor. We assume that *d *is large enough because the number of subtrees is more than the number of processors, that is, one processor executes the enumeration for at least one subtree. In addition, we assume that *d *is not too large so that execution time of generating the vertices with up to depth *d *of a family tree can be ignored. However, the number of generated molecular trees in such a subtree of a family tree often varies a lot. Therefore, we develop three types of assignment methods in BfsEnumP1-3. BfsEnumP1 and BfsEnumP2 take static assignment methods, whereas BfsEnumP3 takes a dynamic assignment method depending on the computational environment during execution. We perform computational experiments for C_26_H_54_, C_16_O_4_H_34_, and C_10_N_3_O_2_H_25 _with varying the division depth *d *and the number of processors, and show that BfsEnumP1-3 are useful to reduce the execution time for enumeration of tree-like chemical compounds. In addition, we show that BfsEnumP3 achieves more than 80% parallelization efficiency using up to 11 processors, and reduce the execution time using 12 processors to about 1/10 of that by BfsSimEnum.

## Preliminaries

### Enumeration problem

A molecular tree can be represented as a rooted ordered tree *T *(*V, E*) with a set *V *of vertices and a set *E *of single and multiple edges, where each vertex corresponds to an atom, and each edge corresponds to a covalent bond. Let Σ = {*l*_1_, *l*_2_,..., *l_s_*} be a set of labels representing distinct atoms. Let *val*(*l_i_*) be the valence of the atom corresponding to *l_i_*. Let *num_T _*(*l_i_*) be the number of vertices labeled as *l_i _*in *T *. Let *parent*(*v*) denote the parent vertex of *v*. Let *l*(*v*) and *degree*(*v*) be the label, and degree of vertex *v *in *T *, respectively. Then, we define the tree-like chemical compound enumeration problem as follows.

**Problem 1 ***Given a set *Σ *of labels, the valence val*(*l_i_*) *and number n_l_i__ of each label l_i_, enumerate all molecular trees T without any redundancy such that num_T _*(*l_i_*) = *n_l_i__ for all l_i _*(∈ Σ) *and degree*(*v*) = *val*(*l*(*v*)) *for all v *(∈ *T*).

### Family tree

**Figure 1:**
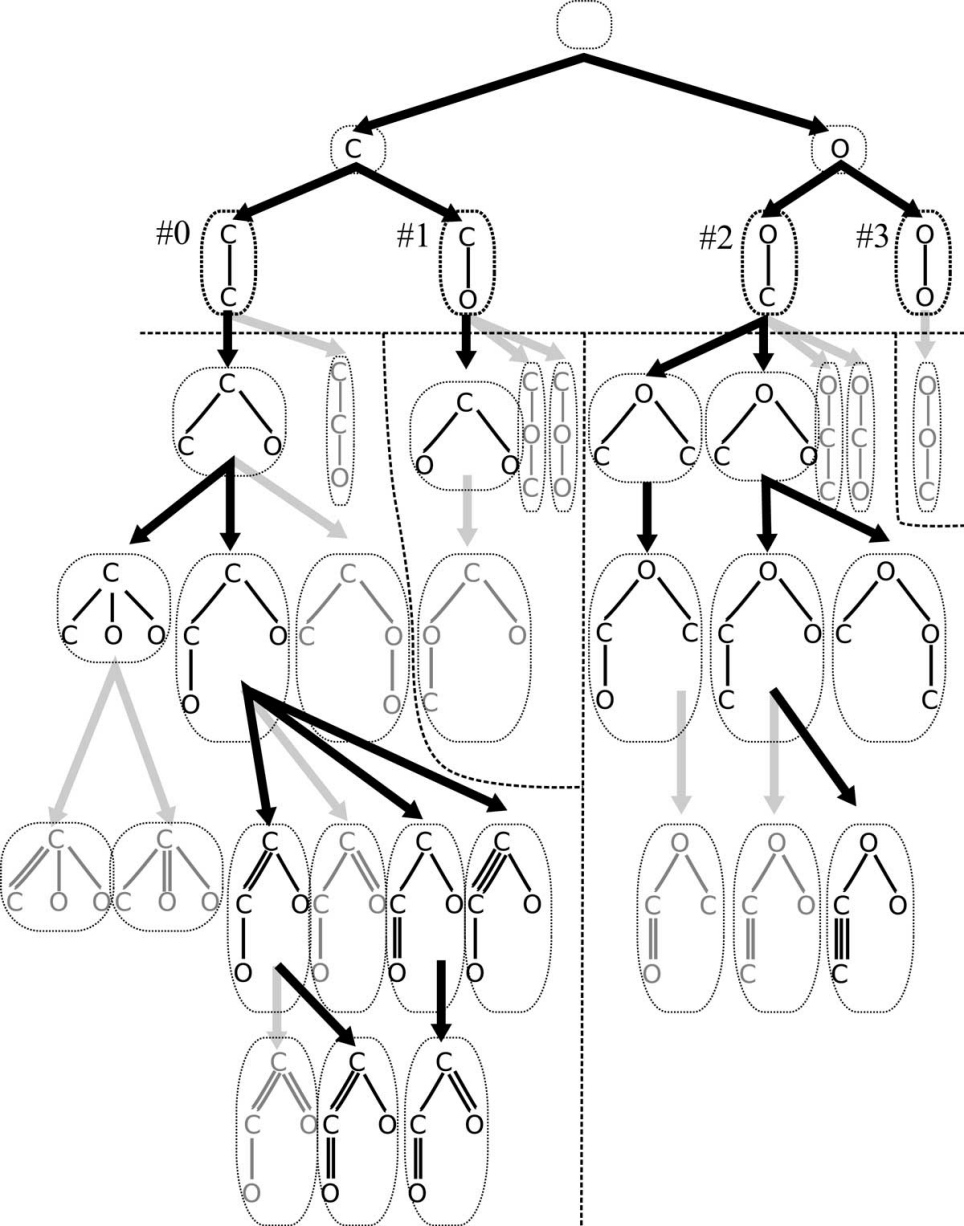
**Example of a family tree by BfsSimEnum and BfsMulEnum for C_2_O_2_H_2 _and its separation by BfsEnumP1-3 with division depth 2**. Molecular trees in gray color are regarded as invalid by the algorithms. It should be noted that hydrogen atoms are added as leaves at last.

Our approach searches a special tree structure called *family tree*, in which each vertex corresponds to a molecular tree. The root is an empty tree. A family tree is grown by adding an atom to some vertex. Figure 1 shows a family tree for C_2_O_2_H_2 _by BfsSimEnum and BfsMulEnum, where hydrogen atoms are added at the end of enumeration. In this example, BfsSimEnum constructs the family tree up to depth 4 by adding atoms, and BfsMulEnum constructs the rest by adding multiplicity to edges.

In the previous study, to reduce the search space (i.e., the size of a family tree), we utilized two constraints for molecular trees, *center-rooted*, and *left-heavy *[6]. Bfs-SimEnum outputs only center-rooted and left-heavy molecular trees. If a molecular tree that does not satisfy both properties of center-rooted and left-heavy is generated in a family tree, it is eliminated. Thus, the size of a family tree is reduced. We call a molecular tree *center-rooted *if its root is the center vertex or an endpoint of the center edge of a path with the maximum length, where the path does not include the same vertex more than once.

We introduce a total order to Σ, for example, C > N > O > H for Σ = {*C, N, O, H*}, and two inequalities >*_s _*and >*_m _*for rooted and ordered trees. Let *T *(*v*) denote the subtree rooted at vertex *v *in *T *. We call a molecular tree *T left-heavy *if for each vertex *v *(∈ *T *) and its children *v*_1_,..., *v_k _, T *(*v_i_*) ≥_*m *_*T *(*v*_*i*+1_) holds for all *i *= 1,..., *k *− 1. We also say that *T *(*u*) is *heavier *than *T *(*v*) for vertices *u *and *v *if *T *(*u*) >_*s *_*T *(*v*) or *T *(*u*) >_*m *_*T *(*v*) holds. Here, inequalities >_*s *_and >_*m *_are recursively defined as follows. Let *u*_1_, *u*_2_,...,*u_h _*and *v*_1_, *v*_2_,..., *v_k _*be the children of *u *and *v*, respectively. We define *T *(*u*) >_*s *_*T *(*v*) if *l*(*u*) >*l*(*v*) holds, or *l*(*u*) = *l*(*v*) and there exists *i *such that for all *j *≤ *i T *(*u*_*j*_) = *_s _T *(*v_j _*), and *i *< min{*h, k*}, *T *(*u*_*i*+1_) >*_s _T *(*v*_*i*+1_), or *i *= *k *<*h*. In particular, we recursively define *T *(*u*) = *_s _T *(*v*) if *l*(*u*) = *l*(*v*) and for all *j *≤ *h *= *k, T *(*u*_*j *_) = *_s _T *(*v_j _*) hold.

Let *mul*(*e*) be the multiplicity of edge *e *in *T *. We define *T *(*u*) *>_m _T *(*v*) if *T *(*u*) *>_s _T *(*v*) holds, or *T *(*u*) = *_s _T *(*v*) and there exists *i *such that for all *j ≤ i mul*(*e_j _*) = *mul*(*e′_j _*) and *mul*(*e*_*i*+1_) >*mul*(*e*′_*i*+1_), where *e*_1_, *e*_2_,..., *e_m _*and *e′*_1_, *e′*_2 _,..., *e′_m _*denote the edges in the BFS order in *T *(*u*) and *T *(*v*), respectively. In particular, we define *T *(*u*) =*_m _T *(*v*) if *T *(*u*) =*_s _T *(*v*) and for all *j ≤ m, mul*(*e_j _*) = *mul*(*e′_j_*) hold.

Then, BfsSimEnum always generates left-heavy and center-rooted trees with labeled vertices to reduce the search space. Finally, a generated molecular tree is discarded if it is not in normal form [6]. We say that a molecular tree *T *is in *normal form *if *T *is center-rooted and left-heavy, and the center of *T *is a single vertex, or the center is an edge (*r, v*) and *T *(*v*) *≥_m _T_v _*(*r*) holds, where *r *is the root of *T *, and *T_v _*(*r*) denotes the subtree rooted at *r *obtained by subtracting *T *(*v*) from *T *. It is proved that if two rooted and ordered trees are different in normal form, these trees represent distinct molecular trees. It should be noted that molecular trees themselves are generated by BFS order while a family tree having molecular trees as vertices is searched by DFS order.

## Methods

We propose three parallelized algorithms BfsEnumP1-3 for enumeration of tree-like chemical compounds by modifying BfsSimEnum in simple manners. Let *N *be the number of processors. In growing a family tree, BfsSimEnum adds an atom to a molecular tree by BFS order. BfsEnumP1-3 take a parameter *d*, grow a family tree up to depth *d *as BfsSimEnum does, and assign numbers to the vertices (molecular trees) in depth *d *by BFS order. Figure 1 shows an example of the family tree for C_2_O_2_H_2 _and numbers, #0, ..., #3, in depth 2. All *N *processors independently construct the family tree up to depth *d *and assign numbers one by one. Each vertex in depth *d *is assigned to exactly one processor, and the processor generates its descendants, the subtree rooted at the vertex of the family tree. However, we observe that the number of generated molecular trees in the descendants is often different. In the example of Figure 1, the number of generated molecular trees for vertex '#0' is eight, and on the other hand, that for '#1' is one. Hence, we develop three types of assignment methods in BfsEnumP1-3 for the sake of distributing the load equally to each processor. BfsEnumP1-2 take static assignment methods, and BfsEnumP3 takes a dynamic method depending on computational environment during execution.

By modifying the previous single algorithm BfsSimEnum, we propose the following parallelized algorithm.

**Input**: numbers *n_l_i__* of atoms for *l_i _*(*∈ *Σ), division depth *d*, processor identifier *p*, number *N *of processors,

$$n_{a}:=\sum_{\left\{l_{i}\in \sum |val\left(l_{i}\right)>1\right\}}{n_{l}}_{{}_{i}},d<n_{a}$$

**Output**: all molecular trees in normal form

**BfsEnumP**(*p, N *)

   *c *:= 0

   **for **each *l_j _∈ *Σ such that *val*(*l_j _*) *>*1, $n_{l_{j}}$ > 0 **do**

      *T *:= a tree consisted of a root with *l_j_*

      AddAtom(*T , p, N *)

end

**AddAtom**(*T , p, N *)

   **if **|*T*| = *n_a _***then**

      **if ***T *is in normal form **then**

         BfsMulEnum(*T *)

   **else**

      *flag *:= *true*

      **if **|*T*| = *d ***then**

         *flag *:= IsAssigned(*c, p, N*)

         *c *:= *c *+ 1

      **if ***flag ***then**

         *v_k _*:= the deepest rightmost vertex in *T*

         *v_l _*:= the deepest leftmost vertex in *T*

         **if ***v_k _*and *v_l _*are included in the same subtree **then**

            *v*_*e *_:= *v*_*l*−1_

         **else ***v_e _*:= *v_k_*

         **for **each *v_i _*from *parent*(*v_k _*) to *v_e _*in BFS order **do**

            **if ***degree*(*v_i_*) *< val*(*l*(*v_i_*)) **then**

            **for **each *l_j _*∈ Σ such that *val*(*l_j_*) *>*1 **do**

               **if ***num_T _*(*l_j_*) <$n_{l_{j}}$**and**

                  *l_j _*does not violate left-heavy **then**

                  *T*′ := *T*

                  add an atom *l_j _*as the last child of *v_i _*in *T′*

                  AddAtom(*T′, p, N*)

end

It should be noted that this pseudocode describes the common part of BfsEnumP1-3, and function 'IsAssigned' provides an assignment method according to BfsEnumP1-3. *c *means the identifier number for each vertex in depth *d *of the family tree. All processors execute the same algorithm with distinct identifier number *p *among *N *processors, and BfsMulEnum(T) sequentially outputs molecular trees by adding multiplicity to edges of *T *if needed. Thus, *N *processors output all tree-like chemical compounds without redundancy.

### BfsEnumP1

We define the assignment method of BfsEnumP1 as follows.

**IsAssigned**(*c, p, N *)

      **return ***p *= *c *mod *N*

end

'IsAssigned' returns whether or not the processor with identifier *p *is assigned to vertex *c*. For instance, in the case of enumeration using 3 processors and division depth *d *= 2 for C_2_O_2_H_2_, vertices 0, 1, 2, 3 are assigned to processors 0, 1, 2, 0, respectively by BfsEnumP1 (see Figure 1).

### BfsEnumP2

We define the assignment method of BfsEnumP2 as follows. First, we initialize weights *w_i _*= 0 for *i *= 0,..., *N *− 1.

**IsAssigned**(*T , p*, {*w_i_*})

      *i *:= argmin_*i *= 0,...,*N *− 1*wi*_

      *w_i _*:= *w_i _*+ *cost*(*T*)

      **return ***p *= *i*

end

In BfsEnumP2, the number of molecular trees generated from *T *is estimated by *cost*(*T *), which is accumulated to *w_i_*. One processor having the minimum of *w_i _*is selected to execute the enumeration from *T *. It should be noted that any communication between processors does not occur during the construction of a family tree as well as BfsEnumP1, and *w_i _*is calculated independently in each processor. In this paper, we define *cost*(*T *) by

$$\begin{array}{c} \underset{v_{i}\in \left\{parent\left(v_{k}\right),\ldots ,v_{k}\right\}}{\sum }val\left(l\left(v_{i}\right)\right)-degree\left(v_{i}\right)\\ +\underset{l_{i}\in \sum }{\sum }c_{l_{i}}\left(n_{l_{i}}-num_{T}\left(l_{i}\right)\right), \end{array}$$

where *v_k _*denotes the deepest rightmost vertex in *T *, and *c_l_i__* is a positive constant for *l_i_*, (*c_C _, c_N _, c_O _, c_H _*) = (1.4, 1.2, 1.0, 0.0). Here the valence of each atom is taken into account. *cost*(*T *) is large if the number of positions that atoms bond and/or the number of remaining atoms are large.

### BfsEnumP3

BfsEnumP3 requires an extra processor to manage the assignment, which receives requests from other processors, and replies an assigned number to each processor. It should be noted that such a manager is not needed if we use shared memory. In this paper, we implement the algorithm using MPI (message passing interface) for avoiding inconsistency of cache memory. On the other hand, processor *p *receives an assigned number as *r *from the manager, and executes the enumeration from vertex *r*.

Finally, BfsEnumP3 in processor *p *sends an end-signal to the manager. Thus, we have the following pseudo-codes.

**Manage**(*N *)

   *globalc *:= 0

   *n_e _*:= 0

   **while ***n_e _< N*

      **if **receive a request from processor *p ***then**

         send *globalc *to *p*

         *globalc *:= *globalc *+ 1

       **else if **receive an end-signal from *p ***then**

      *n_e _*:= *n_e _*+ 1

end

**IsAssigned**(*c, r, p*)

   **if ***c *>*r ***then**

      send a request to the manager

      receive *globalc *as *r*

   **return ***c *= *r*

end

   Here, *r *is initialized as some negative integer.

## Results

For evaluation of our proposed methods, we employed a computer with two Xeon E5 processors under Linux operating system, where hyper-threading was enabled and each processor contains 12 logical processing cores. BfsEnumP1-3 were implemented in C++ using MPI library.

**Figure 2:**
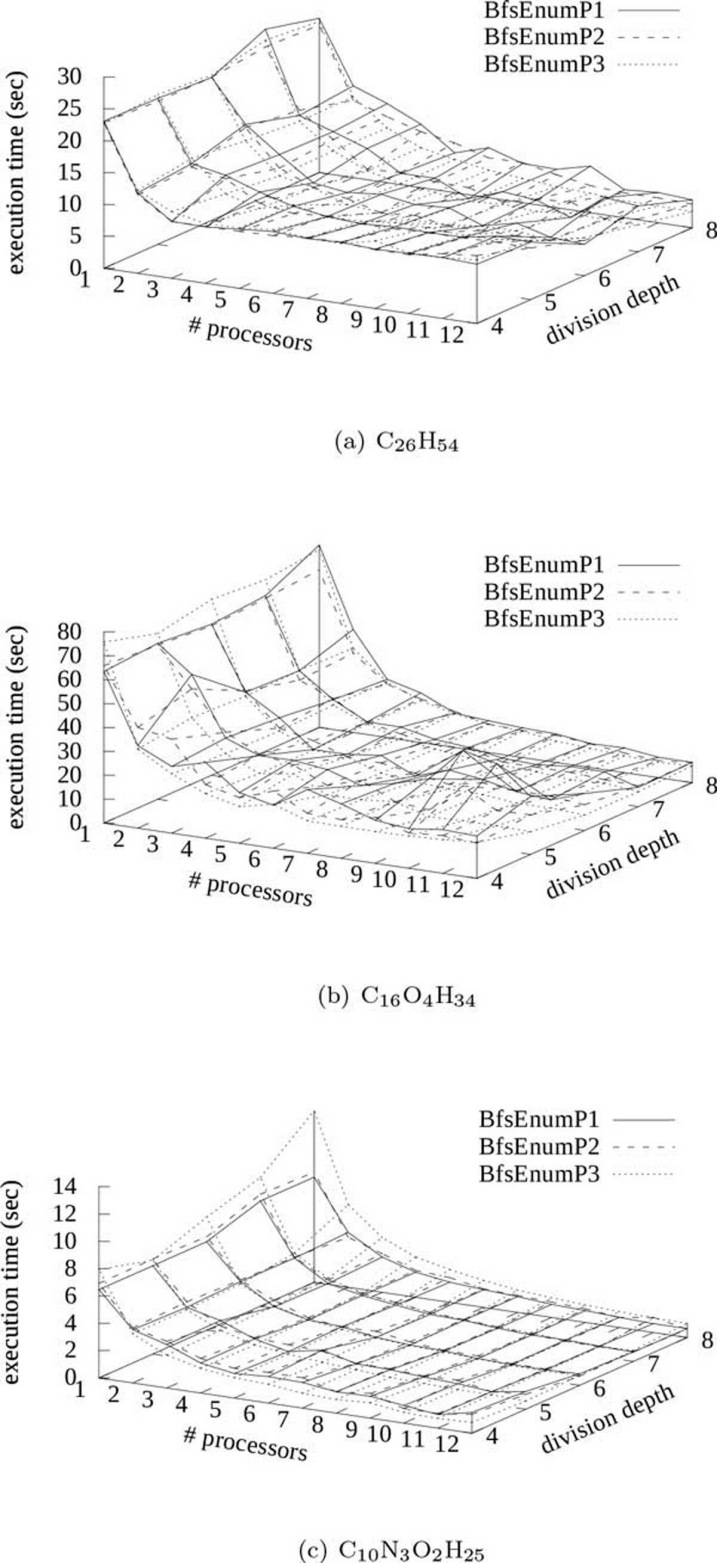
**Result on execution time by BfsEnumP1-3**. 1,..., 12 processors and division depth *d *= 4,..., 8 were examined for (a) C_26_H_54 _(b) C_16_O_4_H_34 _(c) C_10_N_3_O_2_H_25_, where the number of processors for BfsEnumP3 does not include one processor running the manager.

We first examined three instances (*n_C _, n_N _, n_O _, n_H _*) = (26, 0, 0, 54), (16, 0, 4, 34), (10, 3, 2, 25), that is, C_26_H_54_, C_16_O_4_H_34_, C_10_N_3_O_2_H_25_, each of which has only single bonds, using multiple processors. The numbers of enumerated molecular trees for C_26_H_54_, C_16_O_4_H_34_, and C_10_N_3_O_2_H_25 _were 93839412, 278960984, and 29105924, respectively. Figure 2 shows the results on execution times (seconds) by BfsEnumP1-3 with division depth *d *= 4,..., 8 using 1,..., 12 processors for the instances, C_26_H_54_, C_16_O_4_H_34_, and C_10_N_3_O_2_H_25_. We can see that in all cases, the execution time decreased by using multiple processors. For C_26_H_54_, the execution time by BfsEnumP3 with *d *= 8 using 12 processors was 2.54 seconds, which was about 11% of the execution time using one processor. For C_16_O_4_H_34_, the execution time by BfsEnumP3 with *d *= 7 using 12 processors was 6.23 seconds, which was about 10% of the execution time using one processor. For C_10_N_3_O_2_H_25_, the execution time by BfsEnumP3 with *d *= 5 using 12 processors was 0.60 seconds, which was about 9.3% of the execution time using one processor.

In the case of C_26_H_54 _with division depth *d *= 4, the execution times using more than 3 processors were about 9 seconds, and were not reduced unlike in the cases of C_16_O_4_H_34_, and C_10_N_3_O_2_H_25 _(see Figure 2). Hence, we investigated the number of vertices in depth *d *of a family tree.

Table 1 shows the numbers of vertices in depth *d *= 4,...,8 for C_26_H_54_, C_16_O_4_H_34_, and C_10_N_3_O_2_H_25_. The number of vertices in depth 4 for C_26_H_54 _is only 4. It means that if we use more than 4 processors, any task is not assigned to *N − *4 processors. Hence, we need division depth of more than 5 for 12 processors. Furthermore, we can see from the figure that the execution time with larger division depth had tendency to be shorter for C_26_H_54_. On the other hand, the execution time with division depth *d *= 5 was often shorter than others for C_10_N_3_O_2_H_25_. It may suggest that about 1000 vertices in division depth are suitable to be assigned to about 10 processors. If we use larger division depth, we cannot ignore the parallelization overhead. For example, the execution time by BfsEnumP3 with division depth *d *= 8 for C_10_N_3_O_2_H_25 _was longer than those by BfsEnumP1-2 (see Figure 2(c)). It is considered that the overhead of communication between the manager and enumerating processors was large.

**Table 1:** Number of vertices in division depth *d *= 4,⋯,8 of a family tree for C_26_H_54_, C_16_O_4_H_34_, and C_10_N_3_O_2_H_25_.

*d*	C_26_H_54_	C_16_O_4_H_34_	C_10_N_3_O_2_H_25_
4	4	48	282
5	6	138	1026
6	12	379	3844
7	23	1166	14265
8	50	3420	50522

Table 2 shows the results on execution times (seconds) and parallelization efficiencies of BfsEnumP1-3 with division depth *d *= 8 for C_26_H_54_ using up to 12 processors, where one processor for the manager of BfsEnumP3 is excluded. The parallelization efficiency is defined as

**Table 2:** Result on execution time (seconds) and parallelization efficiency of BfsEnumP1-3 with division depth *d *= 8 for C_26_H_54_ using 1,...,12 processors, where one processor for the manager of BfsEnumP3 is excluded.

*N*	BfsEnumP1		BfsEnumP2		BfsEnumP3	
	**time**	**efficiency**	**time**	**efficiency**	**time**	**efficiency**

1	24.20	1.00	23.14	1.00	23.71	1.00
2	14.40	0.84	12.14	0.95	12.72	0.93
3	12.41	0.65	10.35	0.74	8.58	0.92
4	9.97	0.61	8.79	0.66	6.27	0.95
5	6.24	0.78	7.81	0.59	5.19	0.91
6	7.85	0.51	6.24	0.62	4.30	0.92
7	6.15	0.56	6.30	0.52	3.81	0.89
8	6.03	0.50	5.18	0.56	3.32	0.89
9	7.29	0.37	4.26	0.60	3.04	0.87
10	4.42	0.55	3.91	0.59	2.65	0.89
11	4.75	0.46	3.90	0.54	2.65	0.81
12	4.38	0.46	3.73	0.52	2.54	0.78

$$\frac{T_{1}}{N\cdot T_{N}},$$

where *T_N _*denotes the execution time by *N *processors. The execution time by BfsEnumP2 was shorter than that by BfsEnumP1 except using 5, 7 processors. It means that the estimation of the number of generated molecular trees from a vertex worked well for C_26_H_54_. The execution time by BfsEnumP3 was shorter than those by BfsEnumP1-2. Since BfsEnumP2 does not need any communication between processors during the construction of a family tree, BfsEnumP2 can be faster than BfsEnumP3. It, however, is difficult to accurately estimate the number of generated molecular trees. BfsEnumP3 using up to 11 processors achieved more than 80% parallelization efficiency. The parallelization efficiency of BfsEnumP3 decreased especially in using more than 10 processors. It implies that BfsEnumP1-3 using more processors may cause inconsistency of cache memory and decrease the parallelization efficiency. In BfsEnumP1-3, distinct processors do not use the same region of memory. It is considered that if two processors use close regions of memory, inconsistency of cache memory may occur. More processors can decrease the efficiency because the probability of inconsistency increases.

In addition, we examined three other instances (*n_C _, n_N _, n_O _, n_H _*) = (20, 0, 0, 40), (12, 0, 4, 16), (11, 3, 2, 21), that is, C_20_H_40_, C_12_O_4_H_16_, C_11_N_3_O_2_H_21_, each of which includes several multiple bonds. The numbers of enumerated molecular trees for C_20_H_40_, C_12_O_4_H_16_, and C_11_N_3_O_2_H_21 _were 4224993, 282338151, and 7268812476, respectively. In almost all cases using division depth *d *= 4,..., 8 and 1,..., 12 processors, the execution time by BfsEnumP3 was shorter than those by BfsEnumP1-2. For C_20_H_40_, C_12_O_4_H_16_, C_11_N_3_O_2_H_21_, the execution times by BfsEnumP3 with *d *= 8 using 12 processors were 0.0606, 6.93, 83.2 seconds, and 11, 9.1, 9.3 % of the execution time using one processor, respectively. In our previous study, BfsSimEnum and BfsMulEnum were much faster than MOLGEN, and faster or comparable to Enu-Mol. The execution times by BfsSimEnum and BfsMulEnum for C_26_H_54_, C_16_O_4_H_34_, C_10_N_3_O_2_H_25_, C_20_H_40_, C_12_O_4_H_16_, and C_11_N_3_O_2_H_21 _were 22.48, 62.70, 6.61, 0.47, 77.33, and 924.10 seconds, respectively, which were close to those by BfsEnumP1-3 using one processor, and were much longer than those by BfsEnumP1-3 using two processors, respectively.

## Conclusion

In this paper, we proposed three parallelized algorithms BfsEnumP1-3 for enumerating tree-like chemical compounds by modifying our previous method BfsSimEnum. We performed experiments for several instances with varying the parameter of division depth and the number of processors. The execution time by BfsEnumP3 was shorter than those by BfsEnumP1-2 in almost all cases. BfsEnumP3 achieved more than 80% parallelization efficiency using up to 11 processors. In addition, BfsEnumP3 reduced the execution time using 12 processors to about 10% of that by the previous algorithm BfsSimEnum. The results suggest that the division depth should be given so that the number of vertices in the depth is about 1000 for 10 processors.

BfsEnumP1-2 statically assign tasks to processors without communication between processors during the construction of a family tree, whereas BfsEnumP3 dynamically makes the assignment depending on computational environment during execution. BfsEnumP2 can be faster than BfsEnumP3 by accurately estimating the number of generated molecular trees from a vertex in a family tree when we use more processors. The execution time by BfsEnumP2 was not always shorter than that by BfsEnumP1. It is needed to improve the cost function of BfsEnumP2 under the condition that the function is calculated in a very quick way.

It is important to deal with more complex structures including cycles such as benzene and aromatic rings. Extensions toward enumerating general compounds and combination with biological properties should be another future work.

## Competing interests

The authors declare that they have no competing interests.

## Declarations

Publication of this article has been funded by JSPS, Japan (Grant-in-Aid #26240034).
